# Sickness absence and return to work among employees with knee osteoarthritis with and without total knee arthroplasty: a prospective register linkage study among Finnish public sector employees

**DOI:** 10.5271/sjweh.3989

**Published:** 2021-10-31

**Authors:** Leena Kaila-Kangas, Päivi Leino-Arjas, Aki Koskinen, Esa-Pekka Takala, Tuula Oksanen, Jenni Ervasti, Johanna Kausto

**Affiliations:** 1 Finnish Institute of Occupational Health, Helsinki, Finland; 2 Institute of Public Health and Clinical Nutrition, University of Eastern Finland, Kuopio, Finland

**Keywords:** arthritis, cohort study, employment, Finland, musculoskeletal disease, sick leave, work

## Abstract

**Objective:**

This study aimed to examine duration of sickness absence due to knee osteoarthritis (OA) and sustained return to work (RTW) among municipal employees who had at least one compensated sickness absence period due to knee OA. The contribution of sociodemographic characteristics, diabetes and previous sickness absence were assessed. We differentiated between participants with and without total knee arthroplasty (TKA).

**Methods:**

Data from 123 506 employees in the Finnish Public Sector Study were linked with national health and mortality register information. There were 3 231 sickness absence periods (2372 participants) due to knee OA in 2005−2011. Kaplan-Meier curves for sustained RTW were obtained and median time with inter-quartile range (IQR) calculated for those with and without TKA. Cox regression analyses were carried out in multivariable analyses.

**Results:**

The median time to RTW from the beginning of sickness absence was 21–28 days when TKA was not related to sickness absence and 92–145 days when it was. Among participants with no TKA, age 60−64, non-sedentary work, diabetes, and previous sickness absences predicted longer time to RTW, while pain medication predicted a shorter time. Among participants with TKA, non-sedentary work and previous sickness absences predicted a longer time to RTW.

**Conclusions:**

The clinical relevance of the difference in time to RTW between employees with or without TKA was substantial. Employees with knee OA working in physically demanding jobs need work modifications after TKA, and this calls for a dialog between occupational health care professionals and workplaces.

Knee osteoarthritis (OA) is a common chronic disorder that causes pain and functional limitations with difficulties in walking, stair climbing and other lower-extremity tasks. It reduces quality of life (1), work ability, and work participation (2, 3). The etiology of knee OA is not fully understood, but genetic factors, older age, female sex, obesity, injuries, and repetitive loading of the knee joint, eg, frequent squatting, kneeling, and heavy lifting may play a role (4, 5). OA is a complex condition that involves the entire joint, with progressive loss of articular cartilage, synovitis, and changes in the subchondral bone (6). There is often a discrepancy between the severity of symptoms and the severity of knee OA as assessed by radiography, related to individual experience of pain, and the discrepancy can be to either direction (7).

According to a systematic review (8), the prevalence estimates of knee OA have varied considerably depending on the definition. In European studies, the self-reported prevalence has varied between 7.1% and 15.0%. Among Finns aged ≥30 years, the prevalence of clinically assessed knee OA was 6% and 8% among men and women respectively in 2000 (9). The incidence of knee OA is higher among women than men, and it increases with age and increasing relative weight of the population (8). Thus, the occurrence of knee OA is likely to rise due to population aging and the obesity epidemic (10).

The management of knee OA is primarily conservative emphasizing the role of exercise, balance training and weight control (7) in diminishing pain and improving functional capacity (11, 12). Arthroscopic surgery with debridement or partial meniscectomy may not bring long-lasting benefits in pain or physical function (13, 14). In severe cases, total knee arthroplasty (TKA) has been regarded as the best method to reduce pain and disability and maintain physical activity in the long term (15, 16).

According to a review (17), there is limited evidence for a specific symptom threshold that justifies TKA, but the minimum requirements for it are significant, prolonged symptoms with supporting clinical and radiological signs. Obesity, diabetes and smoking, and comorbidities particularly among older patients increase the risk of complications following TKA. On average 15–20% of patients are dissatisfied with their outcome after surgery, usually because of ongoing pain and poor physical functioning (18). Also, low preoperative functioning, comorbidities (diabetes mellitus, pulmonary disease and back pain), psychological distress, or desire to return to high-impact activities and unrealistic expectations have been associated with dissatisfaction following TKA (19).

Most employees return to work after TKA (20–22), but long periods of sickness absence are common (23), and only a minority of patients aged ≥60 years ever return to work (24). A systematic review (25) identified several individual-related factors that were associated with return to work (RTW) after TKA. These included sex, age, educational level, obesity, motivation to return, and physical and mental functioning, as well as work-related factors like physical demands, workplace characteristics, employment status, pre-operative sickness absence and workers’ compensation.

Timely RTW is the primary goal after recovery among working-aged TKA patients, and the recent steep increase in the incidence of TKA (18, 26) has raised concern regarding the consequences of the procedure for RTW and employability (27). In several countries, the current number of TKA patients <65 years is already high, and the figures are expected substantially to rise at least in United States and Europe (27). It is suggested that also the number of revised TKA is rising in those countries (28). According to a meta-analysis (29), the expectable revision rate in ten years has been <5% in general, but 7% among patients aged <60 years.

Ability to work is not defined by only health or health impairment, but also in relation to one’s work demands, and the onset of illness or handicap may not necessarily lead to work disability (30). Because of a lack of research, it is not known whether conservative treatment of knee OA is associated with a shorter or longer time to RTW as compared with TKA. Therefore, we examined time to sustained RTW among Finnish municipal sector employees with sickness absence due to knee OA, using comprehensive register data. We stratified the analyses by treatment modality (TKA or no TKA) and assessed the contribution of morbidity related factors, sociodemographic characteristics, and occupation.

## Methods

### Participants

The study cohort consisted of participants of the Finnish Public Sector study working in ten municipalities and six hospital districts representing about 20% of public sector employees in Finland (31). In the current analyses, we focused on those employees who were working in the target organizations at the start of the follow-up on 1 January 2005 (N=123 506) and had at least one compensated (ie, lasting >10 work days) sickness absence period due to knee OA before the end of follow-up on 31 December 2011. There were 169 330 compensated sickness absence periods (from 65 985 participants) due to any disorder during the follow-up. Of these periods, 3 231 (1.9%) were due to knee OA (among 2372 participants). Data on hospitalization due to TKA in 1996−2004 were explored, and those who had undergone TKA before the study period were excluded from the analyses.

### Data sources

Employers’ registers provided information on socio­demographic factors of the participants. Data on medically certified sickness absence periods were drawn from the national register of the Social Insurance Institution of Finland. The register covers the compensated periods that lasted for >10 workdays including the day when the disability started. Information on hospitalization periods and the surgical procedures targeting knee OA, also on TKA, (recorded according to the Nordic Classification of Surgical Procedures) was received from the Hospital Discharge Register provided by the National Institute for Health and Welfare. Data on reimbursed medication purchases with codes from the Anatomical Therapeutic Chemical (ATC) classification system were retrieved from the Register of Special Reimbursement for Medication Purchases from the Social Insurance Institution of Finland. We obtained data on pensions from the national registers of the Finnish Centre for Pensions, and data on deaths were retrieved from Statistics Finland. Register data were combined by each participant’s personal identification number that is assigned to each citizen of Finland at birth or when receiving a residence permit.

The Ethics Committee of the Hospital District of Helsinki and Uusimaa in Finland approved the study protocol.

### The Finnish work disability compensation scheme

In Finland, all non-retired residents aged 16–67 are eligible for a compensation of incapacity to work due to disorders after the employer period of payment (a minimum of ten days), and thereafter the Social Insurance Institution of Finland compensates sickness absence >10 days. The maximum length of sickness absence compensation is 300 working days per disease in two years. In case of longer-term work disability, a disability pension can be granted either temporarily or permanently.

### Sustained return to work (RTW)

RTW was defined as sustained when it was indicated by the end of sickness absence period that was not followed by a recurrent sickness absence period due to the same diagnosis within 30 days. This was based on the regulations of the Social Insurance Institution of Finland, according to which two consecutive sickness absence periods with the same diagnosis are regarded as one if the working period in between the two absence periods lasted <30 calendar days.

Sickness absence data consisted of the starting and ending dates and diagnostic codes (ICD-10) (32) for all medically certified and compensated sickness absence periods. For the analysis, the duration of each sickness absence period was calculated starting from the initial day of work absence until the end of the compensation period. Sickness absence periods that lasted ≤10 days, and thus were not compensated, were not available in the data. We did not differentiate between partial and full sickness absence days in the analyses.

### Covariates

Covariates were sex, age at the time of sickness absence, and occupation [categorized according to the International Classification of Occupations (ISCO) as follows: managers and professionals (ISCO 1−2), associate professionals and clerks (ISCO 3−4), service and care workers (ISCO 5) and manual workers (ISCO 6−9)]. Region was categorized according to the geographical location of the participant’s work organization, to adjust for possible regional differences in sickness absence practices (data not shown). Health-related covariates were (i) sickness absence lasting >10 days during the previous year due to musculoskeletal disorders (no/yes), and separately due to other disorders (no/yes), respectively (ii), purchased medication for musculoskeletal pain (no/yes) using ATC classification system codes M01 (anti-inflammatory and anti-rheumatic products) and N02 (analgesics) (iii), diabetes (based on entitlement to special reimbursements (no/yes), and (iv) hospitalization periods related to knee OA. Medication purchases and hospitalization periods were recorded for the preceding 30 days and during the following 7 days after the sickness absence period. To adjust for the severity of knee OA related to sickness absence periods with no TKA, we added information on hospitalizations (no/yes) due to knee OA.

### Statistical analyses

The observational unit was a medically certified and compensated sickness absence period (>10 workdays) due to knee OA. We analyzed separately those periods that were associated with TKA and those that were not. The participants could belong only to one of these categories. We excluded sickness absence associated with partial TKA (36 periods).

The Cox proportional hazards model with a cluster option was applied to estimate the time from the beginning of the sickness absence period to RTW. The cluster option considered the intra-individual correlation of sickness absence periods by using the robust sandwich variance estimates in calculating standard errors. In case of hospitalization for TKA, the time to RTW was estimated from the beginning of sickness absence period due to knee OA or from the beginning of the hospitalization period (when there was no previous sickness absence from work due to knee OA).

Hazard ratios (HR) and 95% confidence intervals (CI) were calculated for the time from the beginning of sickness absence to RTW. Observations were right censored in case of disability pension, old age pension, death, or end of the follow-up, whichever came first. Cox proportional hazards models were adjusted for age, sex, occupational group, region, sickness absence during previous year for musculoskeletal disorders, and for other disorders, reimbursed purchase of pain medication, and diabetes. The time until RTW was visually presented by Kaplan Maier curves, separately for those who had undergone TKA and for those who had not. All analyses were performed using SAS software package (version 9.4; SAS Institute, Inc, Cary, NC, USA).

## Results

In this sample of municipal workers, knee OA-related sickness absence periods (N=3 231) comprised 1.9% of all compensated (>10 days) sickness absence periods (N=169 330) and 6.2% of sickness absence periods due to musculoskeletal disorders (N=51 469) between 2005 and 2011. Of the participants with sickness absence due to knee OA, 17.6% (N=416) had undergone TKA.

### Characteristics of participants with sickness absence due to knee osteoarthritis (OA)

Among participants with sickness absence due to OA, the proportion of women was 77.4%, and among those with TKA, 80.1% (table 1). The mean age at the time of the first sickness absence due to knee OA among those with no TKA (N=1956), was 52.8 years (SD 7.2) and with TKA 56.5 years (SD 5.2) at the time of surgery. Seventy-one per cent of those with TKA belonged to the age group of 55 to 64 at the time of surgery. The proportion of participants aged 60−64 was 19.5% among those without TKA and 31.0% among those with TKA. The corresponding figures among manual workers were 33.2% and 21.9%.

**Table 1:** Characteristics of participants.[OA=osteoarthritis]

	All patients (N=2372)		Knee OA patients with no knee arthroplasty (N=1956)		Knee OA patients with knee arthroplasty (N=416)	
		
	N	%	N	%	N	%
Sex						
Men	536	22.6	457	23.4	79	19.0
Women	1836	77.4	1499	76.6	337	80.1
Age (years)						
26–39	116	4.9	114	5.8	2	0.5
40–44	189	8.0	179	9.1	10	2.4
45–49	327	13.8	293	15.0	34	8.2
50–54	517	21.8	442	22.6	75	18.0
55–59	713	30.1	547	28.0	166	39.9
60−64	510	21.5	381	19.5	129	31.0
Occupational group						
Managers and professionals	371	15.6	292	14.9	79	19.0
Associate professionals and clerks	586	24.7	464	23.7	122	29.3
Service and care workers	674	28.4	550	28.1	124	29.8
Manual workers	741	31.2	650	33.2	91	21.9

### Return to work (RTW)

Table 2 presents the adjusted associations between baseline characteristics and RTW when sickness absence due to knee OA was not related to TKA. The median time until RTW was 21–28 days. Sex or hospitalization related to sickness absence due to knee OA were not associated with RTW. Participants aged 60−64 had a lower probability of RTW compared to those aged 26−49 (HR 0.78, 95% CI 0.68−0.88). With managers and professionals as reference, associate professionals and clerks (0.85, 95% CI 0.75−0.97), service and care workers (HR 0.76, 95% CI 0.67−0.86) and manual workers (HR 0.74, 95% CI 0.65−0.85) had a lower probability of RTW. Similarly, sickness absence due to musculoskeletal disorders overall (HR 0.90, 95% CI 0.83−0.98) or due to other disorders (HR 0.87, 95% CI 0.79−0.95) during the previous year, as well as diabetes (HR 0.65, 95% CI 0.53−0.80), decreased the probability of RTW, while purchase of pain medication (yes versus no) shortened it (HR 1.09, 95% CI 1.01−1.18).

**Table 2:** Determinants and time to sustained return to work (RTW) among knee osteoarthritis (OA) patients without knee arthroplasty (N=1956). Fully ^a^ adjusted Cox proportional hazards regression model with a cluster option. Hazard ratios (HR) and 95% confidence intervals (CI).

Baseline characteristics	Sickness absence periods (N=2776)			Days until RTW ^b^ (percentiles)		
	
	N	HR	95% CI	25^th^	50^th^	75^th^
Sex						
Men	651	Ref		15	21	35
Women	2125	1.05	0.95–1.16	15	21	34
Age (years)						
26–49 ^c^	79	Ref		15	21	35
50–54	666	1.03	0.92–1.15	15	21	34
55–59	830	0.91	0.81–1.01	16	23	40
60−64	481	0.74	0.65–0.84	17	26	50
Occupational group						
Managers and professionals	358	Ref		15	21	35
Associate professionals and clerks	630	0.84	0.73–0.96	16	24	43
Service and care workers	816	0.75	0.65–0.86	17	25	49
Manual workers	972	0.74	0.64–0.84	17	26	50
Sickness absence during previous year for musculoskeletal disorders						
No	1708	Ref		15	21	35
Yes	1068	0.90	0.83–0.98	16	23	40
Sickness absence during previous year for other than musculoskeletal disorders						
No	2196	Ref		15	21	35
Yes	580	0.85	0.77–0.94	16	24	42
Hospitalization related to sickness absence due to knee OA						
No	1975	Ref		15	21	35
Yes	801	1.02	0.93–1.12	15	21	34
Reimbursed purchase of pain medication						
No	1214	Ref		15	21	35
Yes	1562	1.12	1.03–1.22	14	20	32
Comorbidity: diabetes						
No	2650	Ref		15	21	35
Yes	126	0.65	0.53–0.80	17	28	61

a Region was also adjusted for.

b Estimated time to RTW when in the reference category of other covariates.

c 26-year-old was the youngest.

Table 3 presents the adjusted associations between baseline characteristics and RTW when sickness absence was related to TKA. The median time until RTW was 92–145 days. Sex had no association, but occupation was strongly associated with time to RTW: while the median time to RTW was 92 days among managers and professionals, the corresponding figures were 108 among associate professionals and clerks (HR 0.51, 95% CI 0.38−0.68 with managers and professionals as reference), 130 among service and care workers (HR 0.34, 95% CI 0.26−0.46), and 145 among manual workers (HR 0.28, 95% CI 0.20−0.39). The occurrence of sickness absence due to musculoskeletal disorders (HR 0.79, 95% CI 0.64−0.97) or other disorders (HR 0.71, 95% CI 0.53−0.94) during the previous year was associated with a longer time to RTW.

**Table 3:** Determinants and time to sustained return to work (RTW) among knee osteoarthritis (OA) patients with total knee arthroplasty (N=416). Fully ^a^ adjusted Cox proportional hazards regression model with a cluster option. Hazard ratios (HR) and 95% confidence intervals (CI).

Baseline characteristics	Sickness absence periods (N=455)			Days until RTW ^b^ (percentiles)		
	
	N	HR	95% CI	25th	50th	75th
Gender						
Men	83	Ref		87	92	108
Women	372	1.00	0.75–1.32	87	92	108
Age (years)						
36–49 ^c^	50	Ref		87	92	108
50–54	80	0.87	0.60–1.28	89	94	115
55–59	180	1.18	0.84–1.66	84	92	100
60−64	141	1.21	0.85–1.72	84	92	99
Occupational group						
Managers and professionals	87	Ref		87	92	108
Associate professionals and clerks	135	0.51	0.38–0.68	92	108	154
Service and care workers	134	0.34	0.26–0.46	94	130	245
Manual workers	99	0.28	0.20–0.39	96	145	325
Sickness absence during previous year for musculoskeletal disorders						
No	280	Ref		87	92	108
Yes	175	0.79	0.64–0.97	89	95	121
Sickness absence during previous year for other than musculoskeletal disorders						
No	374	Ref		87	92	108
Yes	81	0.69	0.52–0.92	90	96	129
Reimbursed purchase of pain medication						
No	177	Ref		87	92	108
Yes	278	1.07	0.86–1.34	86	92	104
Comorbidity: diabetes						
No	436	Ref		87	92	108
Yes	19	0.85	0.63–1.14	89	94	117

a Region was also adjusted for.

b Estimated time to RTW when in the reference category of other covariates.

c 36-year old was the youngest.

**Figure 1:**
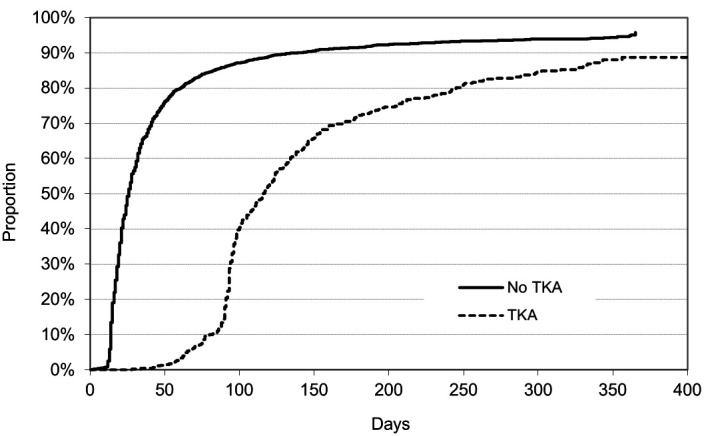
Cumulative proportion of employees with return to work from baseline to 365 days for sickness absence episodes due to knee osteoarthritis with and without total knee arthroplasty (TKA).

Kaplan-Meier curves (figure 1) demonstrate that 92% of the compensated sickness absence periods due to knee OA without TKA ended with RTW within six months, while the corresponding figure among those with TKA was 72%. Within a year, 96% and 89% of the periods, respectively, had ended with RTW.

## Discussion

In this register based, prospective study, we analyzed the time to RTW from the beginning of sickness absence due to knee OA in a cohort of municipal employees who had at least one compensated sickness absence period due to knee OA during the 7-year follow-up. Sustained RTW was defined as the end of the sickness benefit period that was not followed by recurrent sickness absence period for the same diagnosis within 30 days (33). It was assumed that a person was recovered from an acute disorder when she/he had been working 30 days after sickness absence.

Analyses were carried out separately for sickness absence periods (and participants) not related to TKA and for those thus related. At the time of the first sickness absence due to knee OA not related to TKA, the participants were on average 4 years younger than those with TKA, whose mean age was 56 years at the time of surgery.

The median time until sustained RTW was 21–28 and 92–145 days among those without and those with TKA, respectively, when adjusting for sociodemographic characteristics, diabetes and previous sickness absence. Among participants with no TKA, the age of 60−64 years, non-sedentary work, diabetes, and previous sickness absence due to musculoskeletal or other disorders, predicted longer time to RTW, while pain medication predicted a shorter time. Among those with TKA, non-sedentary work and previous sickness absence due to musculoskeletal or other disorders predicted longer time to RTW. A prolonged sickness absence was especially common in service, care and manual work.

The primary strength of our study was the prospective study design and access to comprehensive and valid national register data on sickness absence and hospitalization (34). We could also adjust for many important factors that may have an impact on the time until RTW, including health conditions based on register data on all causes of sickness absence and reimbursed purchases of medication. A cohort of mostly female municipal employees representing hundreds of occupations was followed up. Although municipal occupations involve a large variety of tasks, the results may not be directly generalizable to other branches of industry. Public sector employees are mostly women. Even though the proportion of women was much higher than that of men, the number of men was high enough for comparison. Moreover, the previous studies have shown contradictory sex related results on the time to RTW among TKA patients (20, 35).

There are some limitations in this study. First, we did not have information on short sickness absences. Consequently, the length of sickness absence until RTW may be slightly overestimated. The sickness absence periods related with knee OA comprised 1.9% of all sickness absence periods in our data, in line with a Swedish population-based study that assessed that 2% of all sick days were attributable to knee OA (36). Another limitation is a lack of information on body weight of the participants. There is evidence on obese patients having more difficulties with knee OA and less improvement in TKA outcomes (37). However, diabetes was associated with slower RTW only among sickness absence not related to TKA. This result may be due to health-related selection of individuals to TKA, while diabetes increases the risk of complications following TKA (17). Selection of older patients to surgery or postponement of it due to work-related hindrances can also explain the overrepresentation of those aged 60−64 and the underrepresentation of manual workers among TKA patients.

Most of the previous studies on time to RTW after TKA have based on self-reported retrospective data, and the authors have calculated the full or partial return to work based on the working hours before and after TKA (35). We considered only full return to work. We also measured the time to RTW from the first day of sickness absence and this may have resulted in a longer median time to RTW than in previous studies. In a register-based study, about half of the patients started their sick leave even before the surgery (23).

Lastly, there may have been changes in the care path for OA in Finland after 2005−2011 when the data were gathered. For example, a new law, called the ‘30–60–90-day rule’, was introduced in 2012. This law requires that, after 30 days of sickness absence, the employer needs to inform their occupational healthcare services about the situation of the employee. After 60 days of sickness absence, the occupational healthcare physician needs to assess the need for rehabilitation. And after 90 days of sickness absence, the occupational healthcare physician needs to assess the possibilities of RTW. A register-based study (38) of municipal workers showed, that the effect of the new legislation was most pronounced after about 12 weeks of sickness absence, but the annual gain in work participation was modest.

There is a lack of research on RTW when knee OA is not related to TKA. In a study (39) among Swedish working-age population, the length of the age- and sex-adjusted mean period of sickness absence for knee OA was 81 days. A population-based register study on work ability and work participation of Finnish employees with knee OA (40) found that the median time to RTW was 31 days. However, neither of these studies reported whether sickness absence was related with TKA or not. In a previous study that was based on the same data as the study at hand, the median time to RTW for intervertebral disc disorders (ICD-10: M51) was 32 days among men and 37 among women, and for back pain (ICD-10: M54) 21 and 22 days, respectively (33).

In this study, 92% of the participants with knee OA who did not go through TKA returned to work within six months and about 96% within a year. Half of the sickness absence periods ended with RTW in 21 days. Thus, the median time to RTW was comparable to sickness absence related to back pain in the same population (33). When sickness absence was related to TKA return to work took several months.

According to a descriptive review, the greatest functional improvements occur during the first year after the TKA (41). We found that 72% of sickness absence periods compensated for TKA were associated with RTW within six months, and 89% in one year. The adjusted median time to RTW was 3.1 months among managers and professionals, 3.6 months among associate professionals and clerks, 4.3 months among service and care workers, and 4.8 months in manual work. A systematic review (20) of studies (altogether 649 patients) on RTW after TKA showed that 71–83% of patients were back at work within 3−6 months. In the study of Sankar et al (22), 77% returned to work in 6 months after TKA. Early return to work (within one month) was associated with male sex, university education, working in business, finance or administration, and low physical demands at work. In a prospective study of working patients aged <65 years undergoing TKA for OA, 89% had returned to work one year postoperatively (42). In line with our results, a recent review found that most important factors associated with a slower return to work were a physical nature of employment and preoperative absence from work (35).

We used occupational group as a proxy for physical workload. However, the loads imposed on the knee can vary even within the same job due to individual variation of tasks. It was found (43) that the risk of sickness absence and disability pension due to knee OA was higher in female-dominated job sectors such as health care, childcare and cleaning, which are common among municipal occupations. A large Finnish nationally representative study suggested that 50% of disability retirement due to knee OA among individuals in most manual occupations could be attributed to physical workload factors (44).

A qualitative study (45) reported, that the rehabilitation goals of TKA patients are mainly focused on knee mobility and range of motion, but there is also a need to integrate work into the individually tailored rehabilitation process. However, this requires a dialog between healthcare professionals and employers on appropriate work adjustments (46). Especially occupational health care professionals have knowledge on the work, workplace and potential work adjustments. Obviously, more knowledge is needed on how rehabilitation combined with workplace adjustments can ease RTW and possibly postpone TKA among knee OA patients.

To conclude, our findings showed that 50% of employees on sickness absence due to knee OA returned to work in three weeks when TKA was not needed, and in three months when it was. RTW was especially slow in non-sedentary jobs, and among those with previous sickness absences. Given the burden of knee OA in working age population, the clinical relevance of the difference in time to RTW between employees with or without TKA was substantial, and especially among manual workers. Employees with knee OA working in physically demanding jobs need work modifications after TKA, and this calls for a dialog between occupational healthcare professionals and workplaces.

## References

[1] Vitaloni M, Botto-van Bemden A, Sciortino Contreras RM, Scotton D, Bibas M, Quintero M (2019). Global management of patients with knee osteoarthritis begins with quality of life assessment:a systematic review. BMC Musculoskelet Disord.

[2] Palmer KT, Milne P, Poole J, Cooper C, Coggon D (2005). Employment characteristics and job loss in patients awaiting surgery on the hip or knee. Occup Environ Med.

[3] Sayre EC, Li LC, Kopec JA, Esdaile JM, Bar S, Cibere J (2010). The effect of disease site (knee, hip, hand, foot, lower back or neck) on employment reduction due to osteoarthritis. PLoS One.

[4] Visser AW, de Mutsert R, le Cessie S, den Heijer M, Rosendaal FR, Kloppenburg M (2015). NEO Study Group. The relative contribution of mechanical stress and systemic processes in different types of osteoarthritis:the NEO study. Ann Rheum Dis.

[5] Verbeek J, Mischke C, Robinson R, Ijaz S, Kuijer P, Kievit A (2017). Occupational exposure to knee loading and the risk of osteoarthritis of the knee:a systematic review and a dose-response meta-analysis. Saf Health Work.

[6] Glyn-Jones S, Palmer AJ, Agricola R, Price AJ, Vincent TL, Weinans H (2015). Osteoarthritis. Lancet.

[7] Sharma L (2021). Osteoarthritis of the Knee. N Engl J Med.

[8] Pereira D, Peleteiro B, Araújo J, Branco J, Santos RA, Ramos E (2011). The effect of osteoarthritis definition on prevalence and incidence estimates:a systematic review. Osteoarthritis Cartilage.

[9] Heliövaara M, Impivaara O, Nykyri E, Kaila-Kangas L Hilkka Riihimäki Changes in morbidity. Musculoskeletal disorders and diseases in Finland. Results of the Health 2000 Survey. Publications of the National Public Health Institute, B25/2007.

[10] Zhang Y, Jordan JM (2010). Epidemiology of osteoarthritis. Clin Geriatr Med.

[11] Fernandes L, Hagen KB, Bijlsma JW, Andreassen O, Christensen P, Conaghan PG (2013). European League Against Rheumatism (EULAR). EULAR recommendations for the non-pharmacological core management of hip and knee osteoarthritis. Ann Rheum Dis.

[12] Bannuru RR, Osani MC, Vaysbrot EE, Arden NK, Bennell K, Bierma-Zeinstra SM (2019). OARSI guidelines for the non-surgical management of knee, hip, and polyarticular osteoarthritis. Osteoarthritis Cartilage.

[13] Thorlund JB, Juhl CB, Roos EM, Lohmander LS (2015). Arthroscopic surgery for degenerative knee:systematic review and meta-analysis of benefits and harms. BMJ.

[14] Brignardello-Petersen R, Guyatt GH, Buchbinder R, Poolman RW, Schandelmaier S, Chang Y (2017). Knee arthroscopy versus conservative management in patients with degenerative knee disease:a systematic review. BMJ Open.

[15] Meding JB, Meding LK, Ritter MA, Keating EM (2012). Pain relief and functional improvement remain 20 years after knee arthroplasty. Clin Orthop Relat Res.

[16] Shan L, Shan B, Suzuki A, Nouh F, Saxena A (2015). Intermediate and long-term quality of life after total knee replacement:a systematic review and meta-analysis. J Bone Joint Surg Am.

[17] Adie S, Harris I, Chuan A, Lewis P, Naylor JM (2019). Selecting and optimising patients for total knee arthroplasty. Med J Aust.

[18] Price AJ, Alvand A, Troelsen A, Katz JN, Hooper G, Gray A (2018). Knee replacement. Lancet.

[19] Husain A, Lee GC (2015). Establishing realistic patient expectations following total knee arthroplasty. J Am Acad Orthop Surg.

[20] Tilbury C, Schaasberg W, Plevier JW, Fiocco M, Nelissen RG, Vliet Vlieland TP (2014). Return to work after total hip and knee arthroplasty:a systematic review. Rheumatology (Oxford).

[21] Lombardi AV, Nunley RM, Berend KR, Ruh EL, Clohisy JC, Hamilton WG (2014). Do patients return to work after total knee arthroplasty?. Clin Orthop Relat Res.

[22] Sankar A, Davis AM, Palaganas MP, Beaton DE, Badley EM, Gignac MA (2013). Return to work and workplace activity limitations following total hip or knee replacement. Osteoarthritis Cartilage.

[23] Stigmar K, Dahlberg LE, Zhou C, Jacobson Lidgren H, Petersson IF, Englund M (2017). Sick leave in Sweden before and after total joint replacement in hip and knee osteoarthritis patients. Acta Orthop.

[24] Scott CE, Turnbull GS, MacDonald D, Breusch SJ (2017). Activity levels and return to work following total knee arthroplasty in patients under 65 years of age. Bone Joint J.

[25] Pahlplatz TM, Schafroth MU, Kuijer PP (2017). Patient-related and work-related factors play an important role in return to work after total knee arthroplasty:a systematic review. Journal of ISAKOS:Joint Disorders &Orthopaedic Sports Medicine.

[26] Carr AJ, Robertsson O, Graves S, Price AJ, Arden NK, Judge A (2012). Knee replacement. Lancet.

[27] Kuijer PP, Burdorf A (2020). Prevention at work needed to curb the worldwide strong increase in knee replacement surgery for working-age osteoarthritis patients [Editorial]. Scand J Work Environ Health.

[28] Postler A, Lützner C, Beyer F, Tille E, Lützner J (2018). Analysis of total knee arthroplasty revision causes. BMC Musculoskelet Disord.

[29] Lützner J, Hübel U, Kirschner S, Günther KP, Krummenauer F (2011). Langzeitergebnisse in der Knieendoprothetik :Metaanalyse zu Revisionsrate und funktionellem Ergebnis [Long-term results in total knee arthroplasty. A meta-analysis of revision rates and functional outcome] [German.]. Chirurg.

[30] Mattila-Holappa P, Kausto J, Aalto V, Kaila-Kangas L, Kivimäki M, Oksanen T (2021). Alternative duty work as workplace-initiated procedure to reduce sickness absence. BMC Public Health.

[31] Kivimäki M, Gimeno D, Ferrie JE, Batty GD, Oksanen T, Jokela M (2009). Socioeconomic position, psychosocial work environment and cerebrovascular disease among women:the Finnish public sector study. Int J Epidemiol.

[32] International Classification of Diseases and Related Health Problems (ICD-10), 10th Revision version 2016 World Health Organisation (WHO).

[33] Kausto J, Pentti J, Oksanen T, Virta LJ, Virtanen M, Kivimäki M (2017). Length of sickness absence and sustained return-to-work in mental disorders and musculoskeletal diseases:a cohort study of public sector employees. Scand J Work Environ Health.

[34] Sund R (2012). Quality of the Finnish Hospital Discharge Register:a systematic review. Scand J Public Health.

[35] Van Leemput D, Neirynck J, Berger P, Vandenneucker H Return to work after primary total knee arthroplasty under the age of 65 years:A systematic review. J Knee Surg 2021 Jan.

[36] Hubertsson J, Petersson IF, Thorstensson CA, Englund M (2013). Risk of sick leave and disability pension in working-age women and men with knee osteoarthritis. Ann Rheum Dis.

[37] McGrory BJ, Weber KL, Jevsevar DS, Sevarino K (2016). Surgical Management of Osteoarthritis of the Knee:evidence-based Guideline. J Am Acad Orthop Surg.

[38] Halonen JI, Solovieva S, Pentti J, Kivimäki M, Vahtera J, Viikari-Juntura E (2016). Effectiveness of legislative changes obligating notification of prolonged sickness absence and assessment of remaining work ability on return to work and work participation:a natural experiment in Finland. Occup Environ Med.

[39] Hubertsson J, Englund M, Hallgårde U, Lidwall U, Löfvendahl S, Petersson IF (2014). Sick leave patterns in common musculoskeletal disorders--a study of doctor prescribed sick leave. BMC Musculoskelet Disord.

[40] Kontio T, Viikari-Juntura E, Solovieva S (2020). Effect of Osteoarthritis on Work Participation and Loss of Working Life-years. J Rheumatol.

[41] Canovas F, Dagneaux L (2018). Quality of life after total knee arthroplasty. Orthop Traumatol Surg Res.

[42] Leichtenberg CS, Tilbury C, Kuijer P, Verdegaal S, Wolterbeek R, Nelissen R (2016). Determinants of return to work 12 months after total hip and knee arthroplasty. Ann R Coll Surg Engl.

[43] Hubertsson J, Turkiewicz A, Petersson IF, Englund M (2017). Understanding Occupation, Sick Leave, and Disability Pension Due to Knee and Hip Osteoarthritis From a Sex Perspective. Arthritis Care Res (Hoboken).

[44] Kontio T, Viikari-Juntura E, Solovieva S (2019). To what extent do education and physical work load factors explain occupational differences in disability retirement due to knee OA?A nationwide register-based study in Finland. BMJ Open.

[45] Bardgett M, Lally J, Malviya A, Deehan D (2016). Return to work after knee replacement:a qualitative study of patient experiences. BMJ Open.

[46] Grant M, O-Beirne-Elliman J, Froud R, Underwood M, Seers K (2019). The work of return to work. Challenges of returning to work when you have chronic pain:a meta-ethnography. BMJ Open.

